# Cytotoxicity of Xenogeneic Pericardium Preserved by Epoxy Cross-Linking Agents

**DOI:** 10.17691/stm2021.13.4.03

**Published:** 2021-08-28

**Authors:** N.A. Bondarenko, M.A. Surovtseva, A.P. Lykov, I.I. Kim, I.Yu. Zhuravleva, O.V. Poveschenko

**Affiliations:** Researcher, Cell Technology Laboratory; Research Institute of Clinical and Experimental Lymphology — Branch of the Federal Research Center Institute of Cytology and Genetics, Siberian Branch of Russian Academy of Sciences, 2 Timakova St., Novosibirsk, 630117, Russia; Senior Researcher, Cell Technology Laboratory, Institute of Experimental Biology and Medicine; Meshalkin National Medical Research Center, Ministry of Health of the Russian Federation, 15 Rechkunovskaya St., Novosibirsk, 630055, Russia; Senior Researcher, Cell Technology Laboratory; Research Institute of Clinical and Experimental Lymphology — Branch of the Federal Research Center Institute of Cytology and Genetics, Siberian Branch of Russian Academy of Sciences, 2 Timakova St., Novosibirsk, 630117, Russia; Senior Researcher, Cell Technology Laboratory, Institute of Experimental Biology and Medicine; Meshalkin National Medical Research Center, Ministry of Health of the Russian Federation, 15 Rechkunovskaya St., Novosibirsk, 630055, Russia; Leading Researcher, Cell Technology Laboratory; Research Institute of Clinical and Experimental Lymphology — Branch of the Federal Research Center Institute of Cytology and Genetics, Siberian Branch of Russian Academy of Sciences, 2 Timakova St., Novosibirsk, 630117, Russia; Senior Researcher, Cell Technology Laboratory, Institute of Experimental Biology and Medicine; Meshalkin National Medical Research Center, Ministry of Health of the Russian Federation, 15 Rechkunovskaya St., Novosibirsk, 630055, Russia; Researcher, Cell Technology Laboratory; Research Institute of Clinical and Experimental Lymphology — Branch of the Federal Research Center Institute of Cytology and Genetics, Siberian Branch of Russian Academy of Sciences, 2 Timakova St., Novosibirsk, 630117, Russia; Senior Researcher, Cell Technology Laboratory, Institute of Experimental Biology and Medicine; Meshalkin National Medical Research Center, Ministry of Health of the Russian Federation, 15 Rechkunovskaya St., Novosibirsk, 630055, Russia; Professor, Director of the Institute of Experimental Biology and Medicine; Meshalkin National Medical Research Center, Ministry of Health of the Russian Federation, 15 Rechkunovskaya St., Novosibirsk, 630055, Russia; Head of the Cell Technology Laboratory; Research Institute of Clinical and Experimental Lymphology — Branch of the Federal Research Center Institute of Cytology and Genetics, Siberian Branch of Russian Academy of Sciences, 2 Timakova St., Novosibirsk, 630117, Russia; Head of the Cell Technology Laboratory, Institute of Experimental Biology and Medicine; Meshalkin National Medical Research Center, Ministry of Health of the Russian Federation, 15 Rechkunovskaya St., Novosibirsk, 630055, Russia

**Keywords:** xenopericardium, glutaraldehyde, diepoxide compounds, pentaepoxide compounds, endothelial cells, multipotent mesenchymal stem cells, fibroblasts

## Abstract

**The aim of the study** was to assess the cytotoxic effect of xenopericardial biomaterial treated with di- and pentaepoxides on the cell cultures *in vitro*.

**Materials and Methods:**

Samples of bovine and porcine pericardium were used in the work. Three different modes were employed for preservation: 1) 0.625% solution of glutaraldehyde (GA) and a two-fold change on days 2 and 7; 2) 5% solution of ethylene glycol diglycidyl ether (EGDE) changed on day 2; 3) 5% EGDE solution for 10 days, then 2% pentaepoxide solution also for 10 days. The cytotoxicity of the biomaterial was assessed by the extraction method. To determine the cytotoxicity of the biomaterial, EA.hy926 cells, multipotent mesenchymal stem cells (MMSCs), and fibroblasts were used. Cell viability was determined by the MTT test. The level of apoptosis and necrosis in the cell cultures was assessed by staining with acridine orange and ethidium bromide after cultivation with xenopericardial extracts employing different modes of preservation.

**Results:**

Extracts of bovine and porcine pericardium preserved with GA have been found to have the greatest toxic effect on the cell cultures showing 20–33% reduction of cell viability. Extracts from bovine and porcine pericardium preserved with di- and pentaepoxy compounds do not have a toxic effect on endothelial cells, MMSCs, and fibroblasts since cell viability reduction is by no more than 15%. The lowest level of apoptosis and necrosis is observed in the cell cultures under the influence of extracts from the pericardium, preserved with diepoxide and pentaepoxide compounds.

**Conclusion:**

According to the MTT test for cytotoxicity and determination of the level of apoptosis and necrosis in cell cultures, bovine and porcine pericardia treated with di- and pentaepoxides have been established to have no cytotoxic effect on the culture of endothelial EA.hy926 cells, MMSCs, fibroblasts *in vitro*, whereas GA, in comparison with di- and pentaepoxides, has a toxic impact on the cells.

## Introduction

The search for novel materials for reconstructive and regenerative surgery occupies an important place in biomedical research. The pericardium (bovine and porcine) is widely used in cardiosurgery for prosthetic and plastic repair of cardiac valves and great vessels. When fabricating biografts from xenopericardium, glutaraldehyde (GA) is often used for cross-linking of xenopericardial collagen providing its resistance to biodegradation and prolonged functioning in the patient’s organism. However, it has been shown [1] that GA-fixed biografts may be subjected to calcification in the recipient’s organism. And there exists correlation between the speed and intensity of calcification and patient age: the process runs faster in young patients.

Various agents such as α-amino oleic acid, octanediol, etc., are offered to prevent biograft calcification. In 1987, Nojiri et al. [2] proposed to use epoxy compounds for pericardium cross-linking. It has been shown in the experimental and clinical works that EGDE-fixed bioprostheses possess resistance to calcification, hydrophilic properties, low cytotoxicity, and sterility [3, 4].

Diepoxide-treated biomaterial is not mutagenic and has good functional characteristics [5]. Diepoxide compounds provide increased cross-linking density, better mechanical characteristics, and biological stability of the biomaterial [6]. Polyfunctional epoxide compounds (possessing several epoxide groups) have been proved to enhance these effects compared to bifunctional compounds [7]. All epoxide preservative agents block collagen calcification, although such unambiguous results pertaining to elastin have not been obtained. A pericardial tissue is a completely collagenous material irrespective of the species membership making it possible with a high degree of probability to predict absence of calcification in the biografts made from it using EGDE as a preserving agent.

One of the polyfunctional epoxide compounds is 1,2,3,4,6-penta-O-{1-[2-(glycidyloxy)ethoxy]ethyl-d-glucopyranose (pentaepoxide, PE) containing 5 reactive epoxide groups in the molecule. It was first synthesized at the A.E. Favorsky Irkutsk Institute of Chemistry, Siberian Branch of the Russian Academy of Sciences (Russia) in 1985. The assessment of cytotoxicity of PE-preserved bioprosthetic materials was given in one work only and in regard to only one kind of cells [8].

**The aim of the study** was to assess a cytotoxic effect of xenopericardial biomaterial fixed with di- and pentaepoxides on the cultures of various cells *in vitro*.

## Materials and Methods

### Biomaterials

Bovine pericardia (BP) and porcine pericardia (PP) were harvested from healthy animals at the meat-packing plants. Samples were cleaned from connective tissue, washed several times with 0.9% solution of sodium chloride, and divided in three equal parts for further preservation. Preservation was carried out at room temperature for 6 h after material sampling. Three different materials were used for preservation:

1. GA group: 0.625% solution of glutaraldehyde (Sigma-Aldrich, USA), 0.1 M phosphate buffer, pH 7.4, for 21 h with a two-fold change of the solution on days 2 and 7;
2. EGDE group: 5% solution of ethylene glycol diglycidyl ether (97% purity) (N.N. Vorozhtsov Novosibirsk Institute of Organic Chemistry, Siberian Branch of the Russian Academy of Sciences, Russia), 0.1 M phosphate buffer, pH 7.4, for 14 days changing the solution on day 2;
3. EGDE+PE group: 5% solution of ethylene glycol diglycidyl ether for 10 days, then 2% solution of pentaepoxide (A.E. Favorsky Irkutsk Institute of Chemistry, Siberian Branch of the Russian Academy of Sciences), 0.1 M phosphate buffer, pH 7.4, for 10 days.

### Cell cultures

Endothelial cell line, EA.hy926, was kindly provided by Dr. C.J. Edgel (Caroline University, USA). The cells were cultivated in the DMEM/F12 medium supplemented with 10% fetal bovine serum (FBS) (HyСlone Laboratories Inc., USA), 40 μg/ml gentamicin sulfate (Dalkhimpharm, Russia), and 2 mmol L-glutamine (ICN, USA) in CO_2_-incubator at 37°C and 5% СО_2_ till a confluent monolayer is formed.

The culture of the multipotent mesenchymal stem cells (MMSCs) was obtained from the adhesive mononuclears of the bone marrow from the patients with ischemic heart disease who gave informed consent according to the protocol approved by the Ethical Committee of the Research Institute of Clinical and Experimental Lymphology — Branch of the Federal Research Center Institute of Cytology and Genetics, Siberian Branch of Russian Academy of Sciences (Novosibirsk, Russia). The culture of fibroblast cells FECH-16-2 was acquired at the State Scientific Center of Virology and Biotechnology “Vector” of Rospotrebnadzor (Novosibirsk region, Russia). MMSCs and fibroblasts were cultivated in the DMEM medium supplemented with 10% FBS, 2 mmol L-glutamine, and 40 μg/ml gentamicin. Cells from passages 3–5 were used in the work.

### Assessment of biomaterial cytotoxicity

Toxicity of the biomaterial was assessed using the extraction method in compliance with the standard GOST ISO 10993-5-2011. Extracts of 6 biomaterial groups were prepared for investigation: GA, EGDE, and EGDE+PE of the bovine and porcine pericardium. Extracts were prepared by the method described by Guo et al. [9]. Samples were washed with a sterile buffered physiological solution and weighed. Then they were sterilized by incubation with 70% ethanol for 24 h and washed three times with a sterile buffered physiological solution for 20 min. To obtain extracts, biomaterial samples were cultivated in a complete growth medium for 72 h at 37°С in the ratio of 0.2 g of tissue per 1 ml of medium. After the incubation, supernatants were collected.

To determine cytotoxicity of the biomaterial, EА.hy926 cells, MMSCs, and skin fibroblasts were seeded on the 96-well plates in the quantity of 1**·**10^4^ cells/well. After 24 h of cultivation, the medium was removed and 100 μl of the pericardial extracts were added. Cell viability was tested using colorimetric MTT method (on the basis of 3-(4,5-dimethylthiazol-2-yl)-2,5-diphenyl-2H-tetrazolium bromide) at 24 and 72 h. Absorption of the dissolved formazan crystals was measured at λ=492 nm using a Stat Fax 2100 plate reader (Awareness Technology, Inc., USA).

### Evaluation of apoptosis

To study apoptosis, EА.hy926 cells, MMSCs, and fibroblasts (1**·**10^4^ cells/ well of the 96-well plate) were cultivated for 24 h in the complete growth medium. Then the growth medium was removed and 100 μl of the studied biosample supernatant was added. 24 and 72 h later, the wells were washed two times with a cold buffered physiological solution and 8 μl of acridine orange (100 μg/ml; DIA-M, Russia) and ethidium bromide (100 μg/ml; MEDIGEN, Russia) staining mixture at the 1:1 ratio was added to each well [10]. The cells were visualized by means of Axio Observer microscope (Carl Zeiss, Germany) using a minimum of 500 cells/sample in calculations.

***Statistical analysis*** was performed in Statistica 10.0 program (StatSoft, USA). The results are presented as Me [Q1; Q3]. The data obtained were tested for normality using Kolmogorov–Smirnov criterion. As the distribution in the majority of the experiments differed from normal, the significance of differences was judged by the Mann–Whitney test, considering the differences as significant at р<0.05.

## Results

**Figure 1:**
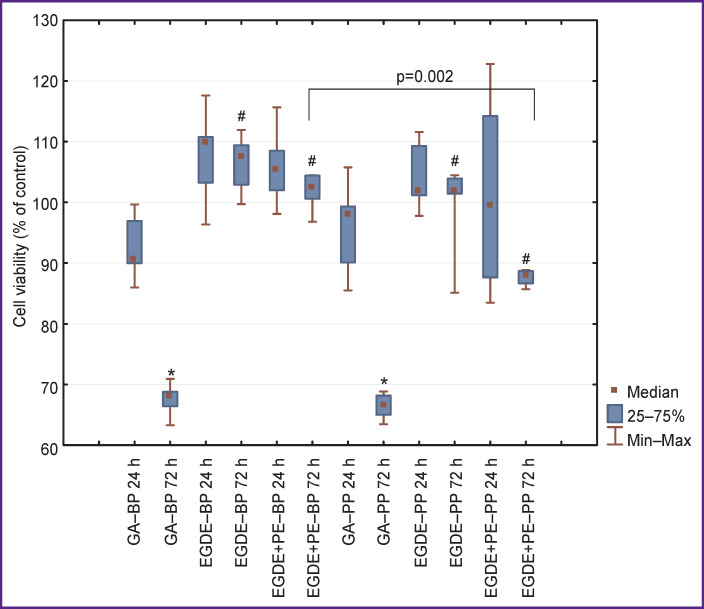
Assessment of toxic effect of xenopericardium treated with GA, EGDE, EGDE+PE on the viability of EA.hy926 cells * р=0.001 for intra-group comparison after 24 and 72 h; ^#^ р<0.01 for comparison between GA group and similar epoxy-preserved groups

On day 1 of observation, the viability of EA.hy926 cells exposed to BP extract treated with GA (GA‒BP group) was statistically significantly (p=0.01) lower relative to the pericardial extract treated with EGDE and EGDE+PE (groups EGDE‒BP and EGDE+PE‒BP) (Figure 1). On day 3, a similar trend was observed. The influence of the BP extracts treated with EGDE and EGDE+PE on the EA.hy926 cells was almost identical during all observation periods: the viability of the cells on day 1 was 110 and 105%, on day 3 — 107 and 102%, respectively.

The viability of EA.hy926 cells exposed to PP extract treated with GA (GA‒PP group) on day 1 was 98%, whereas it was 101 and 99% in the groups EGDE‒PP and EGDE+PE‒PP, respectively. On the whole, this tendency was preserved on day 3 of the experiment. It should be noted, however, that cytotoxicity after the PP treatment with EGDE+PE was statistically significantly higher compared to the BP extract treated by the same method: the viability of EA.hy926 cells in these groups was 88 and 102%, respectively, 72 h later.

**Figure 2:**
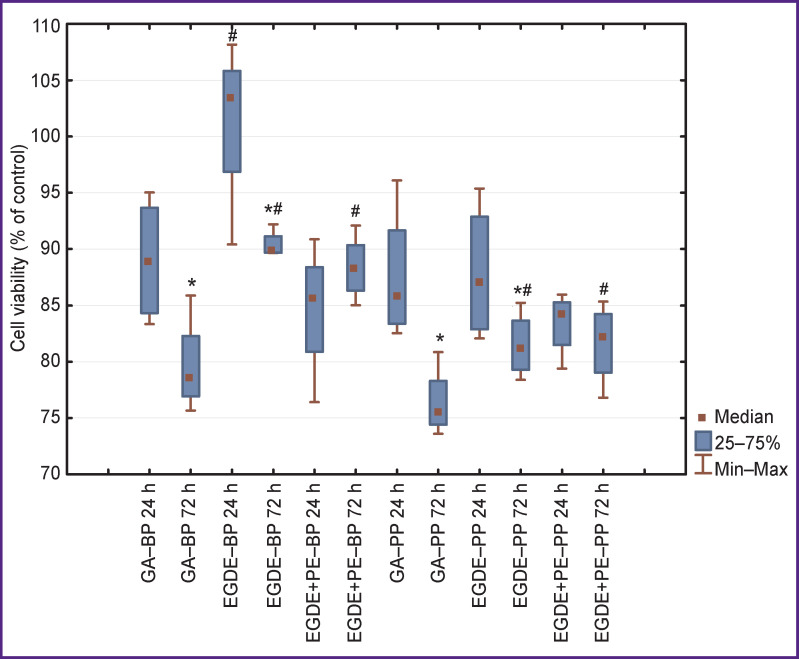
Assessment of toxic effect of xenopericardium treated with GA, EGDE, EGDE+PE preservatives for viability of MMSCs * р=0.003 for intra-group comparison after 24 and 72 h; ^#^ р<0.01 for comparison between GA group and similar epoxy-preserved groups

Much smaller differences in cytotoxicity were observed in the MMSCs depending on the way of the biomaterial treatment. Statistically significantly higher viability of MMSCs on day 1 was revealed only in EGDE‒BP group (Figure 2). MMSC viability on day 3 in the groups EGDE‒BP and EGDE+PE‒BP was statistically significantly higher (р=0.01), i.e. 90 and 88%, respectively, relative to the GA‒BP group (78%). There were no differences in the MMSC viability between the groups GA‒PP, EGDE‒PP, and EGDE+PE‒PP on day 1.

After 72 h, the MMSC viability in the GA‒PP group (75%) was statistically significantly lower (p=0.01) relative to the groups EGDE‒PP and EGDE+PE‒PP: the viability in these groups amounted to 81%. By day 3 of observation, the MMSC viability in the groups GA‒BP, GA‒PP, and EGDE‒BP was statistically significantly lower (p=0.003) in comparison with day 1, whereas the viability in the groups EGDE+PE‒PP and EGDE+PE‒BP on this day was comparable.

**Figure 3:**
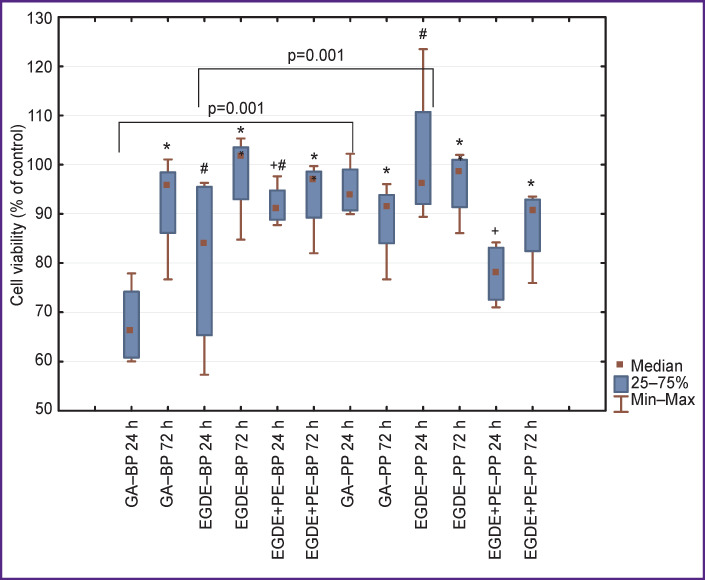
Assessment of toxic effect of xenopericardium extracts treated with GA, EGDE, EGDE+PE preservatives for fibroblast viability * р=0.01 for intra-group comparison after 24 and 72 h; ^#^ р<0.01 for comparison between GA group and epoxy-preserved groups; ^+^ between EGDE and EGDE+PE groups

When studying the effect of pericardium extracts on the viability of fibroblasts, it has been established that after day 1 of observation, the cell viability in the GA‒BP group was statistically significantly (p=0.01) lower (66%) than in the groups EGDE (84%) and EGDE+PE (91%), however these differences have leveled out by day 3 (Figure 3).

Under the action of the GA‒PP and EGDE‒PP extracts, the viability of fibroblasts on the first day was higher (p=0.01) compared to the appropriate BP groups. The viability of fibroblasts exposed to the EGDE+PE‒PP extract was 78% and was statistically significantly lower (p=0.02) than that in the EGDE‒PP group (96%). On the first day of the experiment, the viability of fibroblasts in the EGDE‒BP group was 84% which was statistically significantly lower in comparison with the EGDE+PE‒BP group (91%). By day 3 of observation, the fibroblast viability has leveled out practically in all groups irrespective of species membership of the pericardium and cross-linking agents used for its treatment.

**Figure 4:**
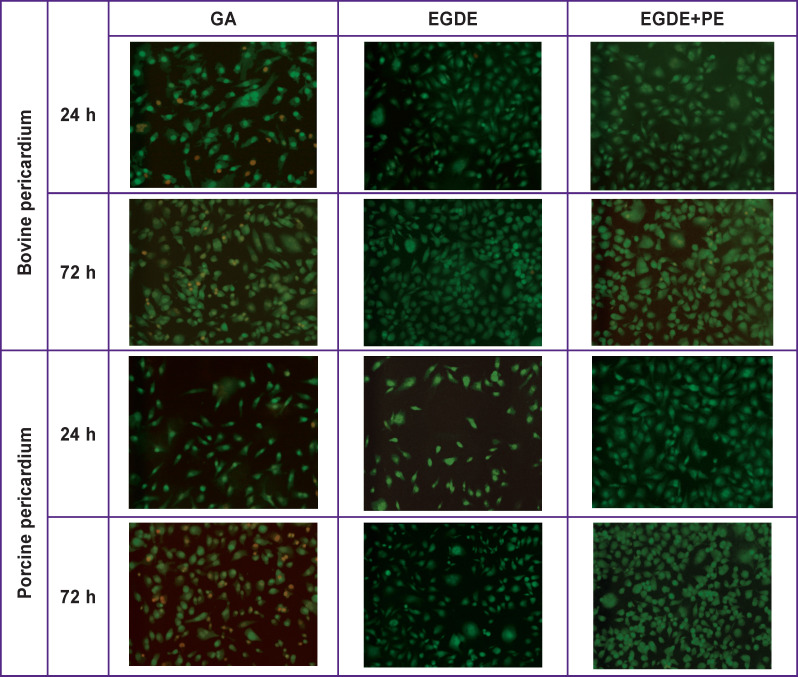
Staining of endothelial EA.hy926 cells with acridine orange + ethidium bromide after addition of BP and PP extracts treated with different preservatives *Green color* — living cells, *red color* — cells at the necrotic stage; ×200

The task of the next stage of the work was to study the effect of extracts from different xenopericardial samples on the level of apoptosis and necrosis of the EA.hy926 cells, MMSCs, and fibroblasts. Statistically significantly larger quantity (p=0.003) of non-viable cells stained with ethidium bromide in red color was found in the groups GA‒BP and GA‒PP relative to the control and other groups: both after 24 and 72 h of observation (Figure 4).

The least significant differences between the values of apoptosis and necrosis level in the EA.hy926 cells were observed in the EGDE+PE‒BP and EGDE‒PP groups on the first day of observation (see the Table). After 72 h, the greatest level of apoptosis and necrosis among all the groups was revealed in the GA‒PP group. As for the cytotoxic effect of the epoxy-treated BP on the EA.hy926 cells, the level of apoptosis was lower in the EGDE+PE group than in the EGDE group after 3 days of observation.

**Table:** The level of apoptosis and necrosis in the EA.hy926 cells, fibroblasts, MMSCs under the influence of pericardium extracts treated with GA, EGDE, EGDE+PE (%) (Me [Q1; Q3])

Cells	Control		Groups													
			GA–BP		GA–PP		EGDE–BP		EGDE–PP		EGDE+PE–BP		EGDE+PE–PP	
EA.hy926	***Day 1***													
	А	N	А	N	А	N	А	N	А	N	А	N	А	N
	0	0	21.5 [6.0; 83.0]*^#^	13.0 [4.0; 23.0]^#^^*	1.1 [1.1; 1.4]	2.2 [1.1; 1.4]^#^	4.2 [0; 15.0]	0 [0; 1.5]*	0 [0; 1.0]	0 [0; 4.0]	0 [0; 1.9]	0 [0; 0.74]	0.8 [0.4; 1.9]	0 [0; 0.4]
	***Day 3***													
	3.7 [3.3; 5.4]*	9.2 [2.7; 16.0]*	0.5 [0.4; 0.9]^	5.2 [2.3; 6.0]^#^	9.2 [5.8; 11.0]*^#^^	10.0 [5.5; 20.0]*^#^	3.3 [2.6; 3.6]	0 [0; 0.6]*	1.4 [0.4; 4.2]*	0.8 [0; 2.3]*	0.7 [0.6; 1.0]^#^	2.3 [2.1; 5.4]*^+^	1.7 [0.4; 3.0]	0 [0; 0.4]
Fibroblasts	***Day 1***													
	0	0	5.1 [3.2; 5.3]^	1.0 [0; 5.0]	1.0 [0.7; 1.1]^	0	2.6 [1.1; 17.0]^+^	0	0 [0; 0.4]	0 [0; 0.6]	1.1 [0; 2.2]	0	0.6 [0.4; 0.6]	0.4 [0; 0.6]
	***Day 3***													
	1.7 [1.6; 1.9]*	0	2.0 [0; 5.0]	28.6 [9.9; 69.0]*^#^	1.2 [0; 1.6]	6.6 [4.0; 6.6]*^#^	1.8 [1.0; 3.0]^+^	2.3 [1.6; 2.3]*^#^	0.5 [0.3; 0.9]^+^	0 [0; 0.5]^+^	2 [0.9; 3.0]	0.1 [0; 0.4]	1.8 [0.6; 5.2]*	0.2 [0; 0.4]
MMSCs	***Day 1***													
	0 [0; 2.9]	0	0 [0; 0.3]	1.6 [1.1; 2.5]	0 [0; 0.3]	1.4 [1.2; 1.6]	0	1.6 [1.1; 2.2]	0.4 [0.1; 0.7]	1.5 [0.9; 1.9]	0	0.8 [0.7; 0.9]	0.1 [0; 0.5]	1.6 [1.5; 1.8]
	***Day 3***													
	0.3 [0; 0.7]	0 [0; 1.6]*	1.8 [1.2; 2.4]*^	2.1 [1.0; 2.9]^#^	0.8 [0.6; 1.2]^	2.9 [2.5; 3.3]*^#^^	1.6 [0.5; 1.9]*	0.9 [0.8; 1.1]	1.5 [1.4; 1.8]*	1.5 [1.4; 1.8]	1.2 [0; 2.4]*	0.8 [0.3; 0.9]	0.6 [0.3; 1.6]	1.1 [0.9; 2.3]

Note: А, apoptosis; N, necrosis; GA, glutaraldehyde; EGDE, ethylene glycol diglycidyl ether; EGDE+PE, ethylene glycol diglycidyl ether and pentaepoxide; BP, bovine pericardium; PP, porcine pericardium. Statistical significance of differences: ^*^ р=0.001 for intra-group comparison after 24 and 72 h; ^#^ р<0.03 for comparison between similar GA and EGDE groups; ^+^ p=0.02 for comparison between EGDE and EGDE+PE groups of bovine and porcine pericardia; ^^^ p=0.01 for comparison between the groups: control and GA–BP, control and GA–PP.

When studying the influence of various extracts on fibroblasts, the level of apoptosis in them was established to be significantly higher (p=0.02) after 1 day in the GA‒BP and GA‒PP groups compared to the control. Besides, in the EGDE‒BP group the level of apoptosis in fibroblasts was statistically significantly higher (p=0.02) compared to the EGDE‒PP group both after 24 and 72 h of observation. Statistically significant differences in the apoptosis level in other groups in these periods were not observed. The groups did not have significant differences in the level of necrosis in fibroblasts on day 1. The level of necrosis in the GA‒BP group was statistically significantly higher (p=0.03) relative to the EGDE‒BP and EGDE+PE‒BP groups. The level of necrosis in fibroblasts in the GA‒PP group was also statistically significantly higher (p=0.02) in comparison with the groups EGDE‒PP and EGDE+PE‒PP. When these groups were compared in the time interval, it was established that the level of apoptosis in fibroblasts by day 3 grows statistically significantly (p=0.001) in the control, EGDE+PE‒BP and EGDE+PE‒PP groups, while the level of necrosis increases in the GA‒BP and GA‒PP groups.

MMSCs appeared to be more tolerant to the xenopericardium extract effect. On day 1 of observation, the level of MMSC apoptosis in the control and experimental groups was comparable. By day 3, it increases significantly (p=0.01) in the GA‒BP and GA‒PP groups relative to the control. The level of MMSC necrosis on day 1 of the experiment grew statistically significantly (p=0.007) in the GA‒BP and GA‒PP groups compared to the control. By day 3, the level of MMSC necrosis increases (p=0.02) in the GA‒PP group relative to the control, GA‒BP, EGDE‒PP, and EGDE+PE‒PP groups. The lowest level of apoptosis was noted in MMSCs in the EGDE+PE‒PP group compared to other preservative agents.

## Discussion

The cytotoxic effect of the BP and PP sample extracts preserved with GA, EGDE, and EGDE+PE on the EA.hy926 cells, MMSCs and fibroblasts has been assessed in the present study. The results have shown that GA-treated extracts of BP and PP reduce the viability of the EA.hy926 cells, MMSCs, and fibroblasts. This allows us to assert that GA-treated extracts of BP and PP possess cytotoxic effect on all types of the studied cells. It should be noted that both kinds of pericardium treated with GA exert a comparable cytotoxic effect on the EA.hy926 cells and MMSCs whereas BP extracts have greater toxic effect on fibroblasts than PP extracts in the first 24 h. The cytotoxic effect of GA-treated BP and PP is associated with free GA remaining in the biomaterial after washing and also with the aldehyde groups which are left unbound due to the masking effect during covalent binding of the cross-linking agent to the biomaterial [11–14].

It has been established that BP and PP extracts preserved with EGDE and the combination of di- and pentaepoxide compounds do not cause considerable toxic effect on the cell of EA.hy926 line, MMSCs, and fibroblasts since the viability of these cells is preserved at the level of the control or decreases, on the average, by 15% under the action of di- and pentaepoxide cross-linking agents.

To identify the mechanisms of cytotoxic effect of BP and PP extracts, the level of necrosis and apoptosis in the cell cultures has been investigated. The lowest level of apoptosis and necrosis was noted in the cells exposed to the biomaterial extracts preserved with epoxide compounds. The data obtained by us agree with the information of the authors [11] who showed that the cross-linking of the collagen materials with epoxides results in the lower cytotoxicity than their treatment with GA.

BP and PP extracts preserved with GA reduce the viability of the cell cultures compared to di- and pentaepoxides. By the end of the observation period, the level of apoptosis caused by the BP extract treated with GA appeared to be higher than in other groups. The obtained data about the influence of GA on the level of apoptosis and necrosis in the cells is also in line with the results of the previous investigations [14, 15], which have demonstrated that not only aldehydes covalently bound to the tissue are found in the GA composition but adsorbed aldehydes as well promoting greatly the reduction of viability of the endothelial cells. It should be noted that the level of necrosis in fibroblasts increased several times under the influence of GA. The data obtained by us agree with the results of the study in which the direct cytotoxic effect of GA on fibroblasts *in vitro* as well as the toxicity of the GA-treated BP extract according to the MTT test have been established [14].

## Conclusion

Assessment of cytotoxicity, determination of the level of apoptosis and necrosis have demonstrated that bovine and porcine pericardia treated with di-and pentaepoxides are not toxic or possess very low cytotoxicity toward the endothelial cells of the EA.hy926 line, multipotent mesenchymal stem cells, and fibroblasts *in vitro*, whereas glutaraldehyde shows a marked cytotoxic effect.
